# Revisiting “Excited Delirium”: Does the Diagnosis Reflect and Promote Racial Bias?

**DOI:** 10.5811/westjem.2022.10.56478

**Published:** 2023-01-31

**Authors:** Brooks Myrick Walsh, Isaac K. Agboola, Edouard Coupet, John S. Rozel, Ambrose H. Wong

**Affiliations:** * Bridgeport Hospital, Yale-New Haven Health System, Department of Emergency Medicine, Bridgeport, Connecticut; † University of Chicago, Northshore University Health System, Department of Emergency Medicine, Chicago, Illinois; ‡ Yale School of Medicine, Department of Emergency Medicine, New Haven, Connecticut; § University of Pittsburgh School of Medicine, Department of Psychiatry, Pittsburgh, Pennsylvania

## Abstract

**Introduction:**

“Excited delirium” (ExD) is purported to represent a certain type of agitated state that can lead to unexpected death. The 2009 “White Paper Report on Excited Delirium Syndrome,” authored by the American College of Emergency Medicine (ACEP) Excited Delirium Task Force, continues to play a pivotal role in defining ExD. Since that report was produced, there has been an increasing appreciation that the label has been applied more often to Black people.

**Methods:**

Our aim was to analyze the language of the 2009 report, the role of potential stereotypes, and the mechanisms that may potentially encourage bias.

**Results:**

Our evaluation of the diagnostic criteria for ExD proposed in the 2009 report shows that it relies on persistent racial stereotypes: eg, unusual strength, decreased sensitivity to pain, and bizarre behavior. Research indicates that use of such stereotypes could encourage biased diagnosis and treatment.

**Conclusion:**

We suggest that the emergency medicine community avoid use of the concept ExD and that ACEP withdraw implicit or explicit support of the report.

## INTRODUCTION

As emergency physicians, we want our patients to be treated humanely, and we want our staff and ourselves to end our shifts healthy and uninjured. Evaluation of the patient with severe agitation or “excited delirium” (ExD) requires us to carefully balance those goals, given the heightened risk of significant harm to the patient, the healthcare worker, or even both. A large amount of research has been directed at clarifying issues of diagnosis, pathophysiology, and best practices for treatment in these situations. The 2009 “White Paper Report on Excited Delirium Syndrome” authored by the American College of Emergency Physicians (ACEP) Excited Delirium Task Force, is a touchstone of that literature. 1 However, there is an increasing awareness of the evidence that Black men receive the diagnosis of ExD more often than White men, and that Black men labeled as having ExD have a higher mortality than White men: Most recently, a report released by Physicians for Human Rights in March 2022 highlighted these concerns, attracting coverage from national news media. 3

At the same time, the emergency medicine (EM) community, including ACEP, has made equitable treatment of patients a priority, including recognition of the role that implicit bias exerts in EM. 4 A statement from ACEP described the death of George Floyd as a manifestation of a “public health emergency,” 5 and affirmed that “ACEP’s mission includes the promotion of health equity within the communities we serve.” They concluded: “The fate of our nation’s public health and safety lies in the balance, and we demand change.”

Clearly, it is imperative to identify the factors that may be contributing to this disparity in ExD diagnosis and outcomes and seek ways to address them. We raise the possibility that the language used in that influential 2009 ACEP report has contributed to biased application of the diagnosis and inequitable outcomes of Black patients. We analyze that language of the 2009 report, the role of potential stereotypes, and the mechanisms that may potentially encourage bias. We will conclude with suggestions to redress those issues.

### The 2009 and 2021 ACEP Task Force Reports

The 2009 “White Paper Report on Excited Delirium Syndrome” (2009 report) authored by the ACEP Excited Delirium Task Force,^1^ has played a prominent role in the discussion about deaths of people in police custody or in medical care. The report is regularly cited in academic literature and popular media. 6 Although it was not published in a medical journal, the document has been readily available through various outlets. 7

The 2009 report described ExD as “a unique syndrome which may be identified by the presence of a distinctive group of clinical and behavioral characteristics.” Such patients were typically “hyperaggressive with bizarre behavior, … impervious to pain, combative, hyperthermic and tachycardic.” Other key features noted were a “failure to recognize or respond to police presence at the scene …, erratic or violent behavior, [and] unusual physical strength and stamina.” They highlighted the often-fatal course of this syndrome, where “a struggle with law enforcement [is] followed by a period of quiet and sudden death.” The report was explicitly written to inform both medical personnel and law enforcement officers.

A different ACEP task force has since produced the “ACEP Task Force Report on Hyperactive Delirium with Severe Agitation in Emergency Settings” (2021 report). 2 The authors state that their document was not intended as an “update or refutation” of the 2009 report. Nonetheless, the 2009 report is referenced, and literature regarding “excited delirium” is cited throughout, including papers both preceding and subsequent to the 2009 report. The 2021 report was approved by the ACEP board of directors, while the 2009 report carries no similar endorsement.

### A Continuing Lack of Clarity in Defining “Excited Delirium”

The criteria used to identify or diagnose ExD have not been clearly established. Aside from the National Association of Medical Examiners, no other leading medical organizations have adopted ExD as a formal diagnostic entity. The American Medical Association (AMA) and the American Psychiatric Association (APA), by contrast, have opposed recognition of this diagnosis. The APA has noted that ExD is “too non-specific to meaningfully describe and convey information about a person.” 8 Similarly, the AMA has issued a statement that ExD lacks a “clear set of diagnostic criteria.” 9 Lastly, while ACEP is often understood to have “officially recognized” ExD, the College clarifies that the 2009 report was an “information paper that was not officially endorsed by ACEP.” 10

**Population Health Research Capsule**

What do we already know about this issue?

*The controversial diagnosis of Excited Delirium (ExD) is disproportionately applied to Black individuals*.

What was the research question?

*Do criteria in the 2009 ExD report from the American College of Emergency Physicians reflect or promote bias?*

What was the major finding of the study?

*ExD was defined using racial stereotypes, and could reinforce inequitable diagnosis and harm*.

How does this improve population health?

*A rejection of the diagnosis of ExD by emergency medicine organizations could reduce patient injury and death, particularly in Black individuals*.

Aside from formal recognition by medical societies, the literature has not provided a better definition of ExD than was offered in the 2009 report. A number of studies have reiterated the criteria offered by Hall in the 2009 report, including subsequent publications co-authored by Hall herself. 11–13 By contrast, a recent study examining a ketamine treatment protocol for ExD in the emergency department (ED) did not describe the authors’ diagnostic criteria, noting only that there is “no current standardized case definition.” 14 A study of agitation in ED patients remarked that ExD as a medical entity, “remains largely theoretical.” 15 One study examined cases in which a police officer had identified ExD, but the diagnosis was not defined or adjudicated. 16 Given the lack of consensus in defining ExD, it is not a surprise that the terms and criteria from the 2009 report continue to be employed.

### Excited Delirium Is a Health Equity Issue

There are decades of evidence demonstrating that young Black males are disproportionately affected by the label of ExD. Some of this evidence was available to the 2009 task force, with four of the studies cited in their report demonstrating disproportionate rates of diagnosis and mortality in Black individuals with ExD. Two of those looked at deaths in South Florida: Ruttender et al found that individuals who died from ExD vs accidental cocaine overdose were more likely to be Black. 17 Mash et al later found a similar racial disparity in results. 18 Grant et al found that Black individuals constituted 63% of the ExD deaths in custody. 19 Stratton et al looked at deaths in people while they were restrained with wrists and ankles secured together behind the back, and labeled as ExD. 20 They found a numerically equal number of deaths in White and Black people; however deaths in Black individuals were higher relative to the population.

The literature subsequent to the 2009 report has also suggested biased application of ExD. A meta-analysis by Gonin et al concluded that being a Black person was an independent risk factor for death in people labeled with ExD. 21 Not only do Black individuals seem to be at higher risk of death with ExD than White individuals, they are also diagnosed in non-lethal cases at a higher rate. Strote et al found that Black individuals represented 56% of the ExD cases in one city, while only 35% were White. 16

Absent from the 2009 and 2021 reports is a substantive discussion of the potential inequitable application of the diagnosis of ExD to Black individuals, and especially Black men while in police custody or under the care of emergency medical services (EMS) care. This issue was unaddressed in the 2009 report, despite findings known at the time. In contrast with the ACEP reports, the popular press has directed increasing attention to the issue of bias and ExD. News reports critically examined the concept of ExD, including racial aspects, after the diagnosis of ExD was advanced by the legal defense team 22 and the police 23 to explain the deaths of George Floyd and Elijah McClain, respectively. National newspapers have published opinion pieces regarding bias in ExD as well. 24

Despite the medical evidence and the attention in the lay press, the 2021 report only briefly touches on the racial disparity in identification and mortality. The authors cite three of these studies mentioned above, and recognize this disproportionate effect on Black individuals. 16,19,20 The 2021 report notes that it may be the case that “differential assessment occurs because persons of color more frequently have dangerous encounters with law enforcement.” This ambiguous wording emphasizes a central aspect of this issue: It may be that Black men are labeled with ExD more often because they interact with law enforcement more frequently. However, it may also be that these men are more often suspected of being dangerous because of an inherently biased conception of ExD.

Similarly, it could be argued that a disproportionate rate of ExD in Black individuals might be simply explained by a disproportionate rate of use of stimulant drugs (eg, powder and “crack” cocaine, phencyclidine, methamphetamine). However, Black individuals seem somewhat less likely to use cocaine or methamphetamine than White individuals. 25 The 2020 National Survey of Drug Use and Health looked at rates of use of powder and crack cocaine, hallucinogens, methamphetamine, prescription stimulants, and central nervous system stimulants among Black and White individuals. 26 Overall, the rate of use of these drugs in the White population exceeded that in the Black population with regard to lifetime use, or in the prior year and prior month use. For example, the 2020 rate of lifetime use of crack cocaine in the age group 18+ years old was marginally higher in the Black population vs the White population (4.4 % vs 4.1%, respectively). However, the Black population in this same age group had a far lower lifetime rate of methamphetamine use than the White population (2.3% vs 7.3%), and this difference was even more marked for lifetime powder cocaine use (9.6% vs 18.6%). And while the rate of crack cocaine use was marginally higher in the Black population, the absolute number in 2020 of White people 18+ years old reporting lifetime crack use (about 6.5 million people) dwarfs that of the Black population (about 1.3 million).

While Black individuals are not, overall, more likely to use cocaine (powder or crack) than White individuals, they are more likely to be arrested for their use of drugs. 27 This appears to be driven by differential use of powder cocaine by White individuals, frequency of use, and socioeconomic factors. However, while a higher rate of arrests for crack cocaine use in Black individuals might explain the higher rate of ExD diagnosis in that population, this would ignore the role of systemic racial biases leading to higher rates of arrest and public perceptions about drug use and crime. 28,29

Regardless of the rate of drug use in either population, drug use is numerically higher in the majority White population. Despite this, common criteria for ExD have used biased language that reiterate racial stereotypes.

### Racialized Criteria for Diagnosis

We argue that a central problem with the criteria for ExD proposed in the 2009 report is the use of language that elicits and reinforces racial stereotypes. We argue that a central problem with the criteria for ExD proposed in the 2009 report is the use of language that elicits and reinforces racial stereotypes. These stereotypes are particularly notable in three of the diagnostic criteria that the 2009 report employs: “unusual” or “superhuman” strength; reduced sensitivity to pain or “impervious”; and “hyperaggressive” (sic) or “bizarre” behavior.

First, the 2009 report describes the patient with ExD as possessing “unusual” or “superhuman” strength. This is uncommonly subjective language for a medical description, but this criterion has remained a standard element of ExD. Even a recent study (cited within the 2021 report) uses “lack of tiring [or] unusual strength” as inclusion criteria for ExD. 30 However, Black individuals have long been stereotyped as possessing significant physical strength and stamina, especially when compared with White individuals. 31–33 Even when the actual strength of the subject is controlled for, Black men are perceived as stronger than White men. 34 The description of “superhuman” strength has an especially freighted racial history. A significant proportion of Americans implicitly and differentially ascribe superhuman or fantastical qualities, including “superhuman” strength,” to Black individuals. 35

The authors of the 2021 report appropriately avoid reinforcing the term “superhuman,” preferring the term “indefatigability.” They offer that indefatigability is “commonly misinterpreted as ‘superhuman strength,”’ but do not explain how that misinterpretation arose. We do not believe that the characterization of “indefatigability” is sufficiently distinct from “superhuman strength,” and may still promote a racially biased conception of ExD. This distinction may be irrelevant, however, as “superhuman strength” continues to be used as a criterion in recent studies of ExD. 36

Second, the 2009 report describes a decreased sensitivity to pain as a central and common characteristic of individuals with ExD. The authors caution that individuals with ExD may have a characteristic “pain tolerance,” making it more likely that control measures that rely on “pain compliance” may fail. An unfortunate but persistent stereotype is that Black individuals are believed to feel less pain than White individuals. 33,37,38 These beliefs are held not only by lay people, but even by medical students 38 and nurses. 39

The 2009 report describes not just a higher tolerance for pain in individuals with ExD, but an inability to feel any pain whatsoever: the phrase “impervious to pain” is used three times. Troublingly, Black individuals have been stereotyped as possessing just such a supernatural capacity to feel no pain. This “superhumanization” stereotype—that Black individuals may feel less or no pain— has roots in the era of slavery (“What would be the cause of insupportable pain to a white man a Negro would almost disregard” 32) and remains widely held. 35

Lastly, the 2009 report uses behavioral abnormalities as key diagnostic features for ExD: The subject may be “hyperaggressive,” “erratic,” or show “destructive or bizarre” behavior, and may vocalize “guttural sounds.” There is ample literature showing that people view Black individuals as more irrational, animalistic, and dangerous than White individuals. 32–34 Black patients are restrained at a higher rate in EDs than are White patients, suggesting an implicit bias in perception of dangerousness. 40 This bias is even shared by psychiatric workers. 41

These stereotypes of unusual or “superhuman” strength, reduced or “impervious” to pain, and “hyperaggressive” behavior, constitute key features of ExD in the 2009 report. We are concerned that the use of this language may encourage the biased diagnosis and treatment of ExD in two manners. First, this language could preferentially evoke the image of a Black male. The tropes of “superhuman,” “impervious,” and “hyperagressive (sic)” have so long been associated with that population that their use here could lead to implicit association of the diagnosis with Black individuals. This use of Black faces or of stereotypically Black words has been shown to do just this in research settings. “Priming” with subliminal cues (eg, words associated with “Black” words, or a Black individual’s face) can promote racially biased judgments in both police officers 42 or therapists, 43 even in scenarios where race is not explicitly mentioned.

Conversely, being presented with a Black person’s face may trigger biased perceptions. In a research setting, participants were far more likely to assign superhuman strength or pain tolerance to faces of Black people compared to White people. 35 Medical workers are subject to the “representative” heuristic, where certain incidental aspects of a case may lead the unwary clinician to prematurely assign a diagnosis. 44 For example, a study enrolling nurses found they were more likely to ascribe the presentation of chest pain or stroke to a less concerning diagnosis if suggestions of depression or alcohol use were introduced into the scenario.. 45 Even a subliminal exposure to the face of a Black individual, displayed too quickly to be consciously registered, might trigger associations with certain diseases, even in physicians. 46 Given this evidence, it could be the case that EMS workers may be led to apply the label of ExD when presented with a Black patient vs when presented with a White patient. Further studies could address this.

The use of racialized terms and images in the 2009 report do not suggest any conscious or explicit bias on the part of the authors. Healthcare workers can manifest certain biases even in the absence of conscious bias. 47 Furthermore, these biases can be exacerbated in the stressful conditions and time pressures of the healthcare environment. 48–50 Our criticism of the 2009 report should not be misunderstood as an accusation of explicit bias on the part of the ACEP task force members.

### “Just semantics?”

The issues we describe with ExD may strike many as “just semantics,” with concerns resolved through simple substitution of the term. The writers of the 2009 report argued that even if the term of ExD was not accepted by other organizations, other diagnoses “describe the same entity as [excited delirium syndrome], albeit with different wording.” 1 Likewise, a recent controversial presentation prepared by an emergency physician for police training was titled “ Excited Delirium Severe Agitation with Confusion (Delirium),” 51 where the use of the strikethrough suggested that only a superficial name change was needed.

However, issues of semantics, by definition, involve differences in meaning, and this is no less true with diagnostic labels. The authors of the 2009 report note that there were several widely accepted and applicable alternative diagnostic labels available. Nonetheless, they felt that the distinct label of “excited delirium” should be applied, and that the features of “superhuman strength,” “hyperaggressive with bizarre behavior,” and “impervious to pain,” were key elements of that entity. The semantics were important to the authors and remain so now.

The authors of the 2021 report write that this discussion over the term ExD is increasingly irrelevant, as the “increasingly charged term” is less often used in favor of more descriptive terms. Nevertheless, the authors recommend that “robust documentation” of patient death, presumably by emergency physicians or other clinicians, can support the medical examiner in determining whether death was due to ExD. In this manner, far from acting as an update or revision of the 2009 report, the 2021 report reinforces the concept and language of ExD, even as one author of the report states that ExD “is on its way out as a diagnostic term.” 52

Additionally, the term ExD endures in academic literature, 14,30,36 and within police 53 and EMS 54 training materials. As we write this, the mayor of a major city has become involved in a controversy regarding the police force, an academic medical center, and police training materials prepared by emergency physicians using the term “excited delirium.” 51 Lastly, emergency physicians providing expert witness testimony in court continue to authoritatively cite the 2009 report in, for example, depositions for civil cases decided in 2020, 55 2021,^56^ and for a grand jury testimony in 2021. 57 It is reasonable to expect that the 2009 report will remain relevant for some time if not challenged.

### A Constructive Way Forward - Four Actions

#### 1. Emergency medicine should avoid the concept of “excited delirium.”

The discussion above has shown that the conception of ExD has roots in racist language and imagery. There is little medical evidence that supports a distinct entity of ExD, while there is growing evidence that the label is associated with health inequities. Thus, there is no basis to use this label over more established medical diagnoses. And indeed, there is evidence that the term is associated with patient harm. Simply replacing the label ExD with another term would not be sufficient.

Any diagnostic label that relies on criteria emphasized in the 2009 report (eg, “unusual” strength, “impervious to pain, or “hyperaggressiveness”) should be considered to be equivalent to ExD, despite any superficial name changes. 51 The emergency medicine community has already begun to eliminate ExD as a valid medical label. The Colorado Department of Public Health & Environment released a report in December 2021 that could serve as an example of a more equitable approach. 58 The Ketamine Investigatory Review Panel, convened in response to the death of Elijah McClain, was chiefly composed of authors and reviewer experts from EM and EMS. The report rejected ExD as a diagnosis, suggested best practices for identification and treatment of dangerously agitated patients, and called for a research agenda to study inequitable use of prehospital sedation. Similarly, in April 2022 the American Academy of Emergency Medicine issued a position statement that ExD is not currently supportable as a medical diagnosis and should not be identified as a cause of death. 59

We would encourage other EM organizations (eg, the Society for Academic Emergency Medicine, the National Association of EMS Physicians, ACEP, the Canadian Association of Emergency Physicians) to examine the problematic conception of ExD and reject it as a valid diagnosis.

#### 2. Clinicians Should Use Established Medical Diagnoses

We have highlighted that concerns about ExD cannot be addressed by a simple name change. We suggest that standard diagnostic labels be employed. For example, the APA has noted that “Delirium, hyperactive subtype” from their Diagnostic and Statistical Manual (DSM) captures many elements of a patient’s presentation, 8 but without the stereotyped language of ExD. The authors of the 2021 ACEP report use similar language of “hyperactive delirium with severe agitation,” although they do not refer to DSM criteria or provide their own definition. 2 We suggest that clinicians use such a diagnostic structure in lieu of ExD.

#### 3. ACEP Should “Retire” the 2009 Report

ACEP has clarified that the 2009 report was produced as an “information paper” but was not officially endorsed by ACEP (personal communication 10). However, there exists an understanding that ACEP “formally declared” the existence of ExD 60 or that ACEP had “formally recognized” ExD. 61 This language of “formal” recognition by ACEP has been repeated in EM trade publications 62 and even on the ACEP website. 63 While ACEP has not formally endorsed the 2009 report, neither has the college corrected any such mischaracterizations. The 2021 report did not aim to accomplish this, and the authors of that paper were explicit that their work “is de novo and not to be construed as an update or refutation of the 2009 paper.”

We suggest that ACEP formally withdraw acknowledgment of the 2009 report. This should be followed by proactive engagement, to correct mischaracterizations of “formal” or “official” status of the 2009 report. Such efforts would comprise clear communication with editors of academic medical journals, as well as outreach to lay media. Furthermore, emergency physicians working as expert witnesses in civil or criminal litigation should be directed to avoid describing or implying any ACEP “endorsement” or “recognition” of ExD.

#### 4. Consider Greater Professional and Racial Diversity in Future Panels

We should not preclude further efforts to discuss the label of ExD by ACEP or other organizations. It is possible that future evidence could support a distinct diagnostic label. As we have discussed, there are significant issues of bias that complicate the concept of ExD. Thus, a wider range of perspectives need to be represented in a future task force.

First, such a task force of emergency physicians and other stakeholders should include those with expertise in not only EMS and toxicology, but also neurology, emergency psychiatry, and health equity. A wider range of community and advocacy leaders should also be considered. 64 Second, a future task force should include a broader racial perspective. 65,66 The recent release of the Ketamine Investigatory Review Panel Report by the Colorado Department of Public Health & Environment can serve as an example of professional, racial, and ethnic diversity. 58

## CONCLUSION

Emergency medicine and ACEP, specifically, has committed to recognizing and addressing structural racism and working to ensure equitable treatment of patients. Identification and management of the severely agitated patient is a key challenge in EM, with such patients often described as being in a state of excited delirium. The evidence shows, however, that Black people are differentially labeled with ExD, seemingly dying at a higher rate than White people. Despite concerns about the diagnosis of ExD, the 2009 ACEP report on ExD continues to be used and cited as an important resource, viewed by some as an “official endorsement” of ExD by ACEP. We have found that the report uses racialized language and imagery to define ExD and that such framing may encourage biased care of agitated patients. We conclude that emergency physicians should avoid this diagnostic concept, and researchers should adopt more established criteria when studying agitation and delirium. Lastly, we urge that ACEP actively rescind any explicit or implicit endorsement of the 2009 report. This position should be communicated to law enforcement organizations and to expert witnesses testifying in relevant civil and criminal litigation.
